# Pecan (*Carya illinoinensis* (Wagenh.) K. Koch) Nut Shell as an Accessible Polyphenol Source for Active Packaging and Food Colorant Stabilization

**DOI:** 10.1021/acssuschemeng.0c00356

**Published:** 2020-04-13

**Authors:** Federica Moccia, Sarai Agustin-Salazar, Anna-Lisa Berg, Brunella Setaro, Raffaella Micillo, Elio Pizzo, Fabian Weber, Nohemi Gamez-Meza, Andreas Schieber, Pierfrancesco Cerruti, Lucia Panzella, Alessandra Napolitano

**Affiliations:** † Department of Chemical Sciences, University of Naples “Federico II”, Via Cintia 4, I-80126 Naples, Italy; ‡ Institute for Polymers, Composites and Biomaterials (IPCB-CNR), Via Campi Flegrei 34, I-80078 Pozzuoli, Italy; § Institute of Nutritional and Food Sciences, Molecular Food Technology, University of Bonn, Endenicher Allee 19b, D-53115 Bonn, Germany; ∥ Department of Biology, University of Naples “Federico II”, 80126 Naples, Italy; ⊥ Departamento de Investigaciones Científicas y Tecnológicas de la Universidad de Sonora, Rosales y Blvd. Luis Encinas, C.P. 83000 Hermosillo, Sonora, México; # Institute for Polymers, Composites and Biomaterials (IPCB-CNR), Via Previati 1/E, I-23900 Lecco, Italy

**Keywords:** Pecan nut shell, Condensed tannins, Antioxidant, Fruit browning inhibition, Food colorant stabilization, Polylactic acid, Active packaging, Anthocyanins

## Abstract

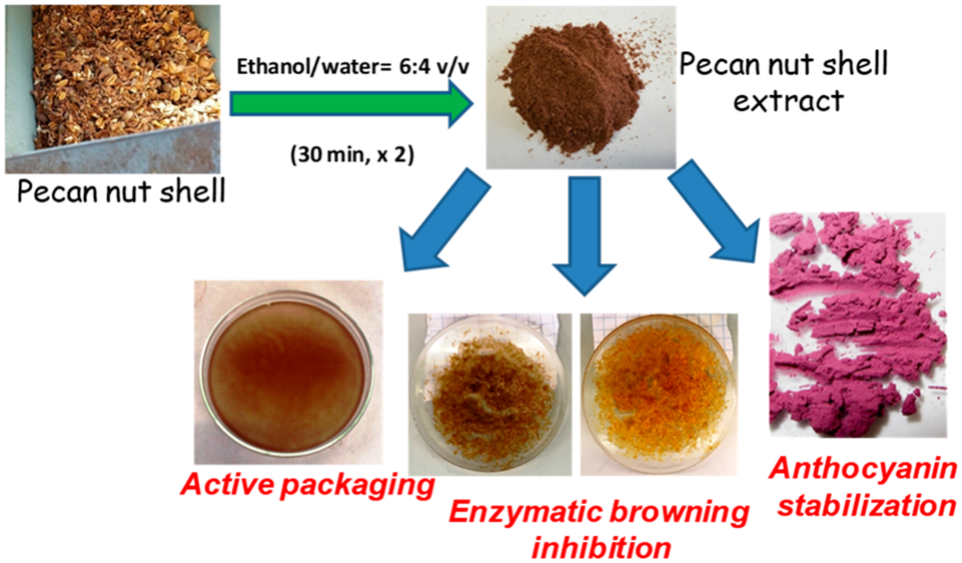

Herein, the antioxidant and food stabilizing properties of a pecan nut shell (PNS) hydroalcoholic extract (PNSE) are reported. Chemical degradation of PNSE demonstrated the presence of condensed tannins as the main phenolic components. PNSE showed remarkable antioxidant properties in the 2,2-diphenyl-1-picrylhydrazyl (DPPH) assay (EC_50_ = 0.004 mg/mL). PNSE was initially tested as an inhibitor of mushroom tyrosinase, exhibiting a quite low IC_50_ value (0.055 mg/mL) against the enzyme diphenolase activity, suggesting its use in enzymatic browning inhibition. The anthocyanin stabilization properties were evaluated under accelerated aging conditions of both pure pigments and commercial fruit juices, and PNSE was found to be effective at concentrations (0.05 mg/mL) at which well-known stabilizers such as chlorogenic and ferulic acids proved to fail. PNSE also performed well in the stabilization of spray-dried anthocyanins for use as a food colorant, increasing the half-life of blackberry anthocyanins up to 20%. In order to explore the possibility of using PNSE as a functional additive for active packaging, polylactic acid (PLA) films containing PNSE were prepared by solvent casting, and no substantial alteration of the mechanical properties was found on addition of the extract up to 10% w/w. The films showed remarkable antioxidant properties (DDPH reduction >60% with a 3% w/w loading, at a dose of 1 mg/mL in the DPPH solution) and delayed the onset of browning of apple smoothies (ca. 30% inhibition with a 10% w/w loading). These results highlight the exploitation of PNS as a low-cost polyphenol source for food industry applications.

## Abstract 2

Pecan nut shell represents an easily accessible and sustainable source of functional additives for food industry applications.

## Introduction

The search for natural and sustainably produced antioxidant additives for use in health, food, or cosmetic applications has received considerable attention in recent years, prompted by the increasing need for green and sustainable approaches to novel functional materials.^1−4^ In this context, waste materials from agrifood industries represent an easily accessible source of phenolic compounds that, apart from their use as food supplements or as additives in functional foods, have become increasingly attractive also from a technological point of view due to their possible exploitation in materials science.^5−9^ Incorporation of antioxidant additives into polymers, both for stabilization and functionalization purposes, is particularly relevant for active food packaging. This latter represents an important sector of the food industry to avoid or delay oxidative deterioration of food.^10−15^ Several approaches have been proposed to provide biocompatible polymers with antioxidant functionality, the most sustainable of which being based on the simple incorporation of the additive by extrusion or solvent casting, without the need for chemicals to covalently link the antioxidant to the polymer.^16−20^ Several articles have reported that polyphenols from agroindustry byproducts, including grape pomace, spent coffee grounds, and orange peels, are able to exert a powerful stabilizing action on several polymers.^6,20−23^ Another noticeable example is represented by pecan nut shell (PNS) hydroalcholic extract (PNSE), which acted as a thermal and photo-oxidative stabilizer of both polyethylene (PE) and polylactic acid (PLA).^24^ Acid insoluble lignins obtained by sulfuric acid treatment of PNS were also found to confer higher ductility to PLA biocomposites determining an increase in stress and strain at break.^25^

The pecan (*Carya illinoinensis* (Wagenh.) K. Koch) nut belongs to the *Juglandaceae* family and is native to the south of the United States and north of Mexico. Pecan nut processing results in the production of a high amount of shells, containing mainly fiber (cellulose, hemicellulose, and lignin) as well as a significant amount of antioxidant phenolic compounds,^24,25^ which are commonly used for infusion in folk medicine due to their beneficial health effects. More recently, the use of PNS as an adsorbent to remove toxic ions and organic pollutants from aqueous solutions^26−28^ and as a reinforcing filler for polymers has been described.^25,29^ The impact of an aqueous extract of PNS on the oxidative stability of margarines during storage has also been reported.^30^ The exploitation of PNS as a carbon source for fungal or bacterial production of bioactives is another emerging field of application of this multifaceted waste material.^31,32^

Prompted by these observations, the possible use of PNSE in food industry applications, with particular reference to antioxidant active packaging, enzymatic browning inhibition in fruits, and stabilization of anthocyanins largely used as food colorants, was investigated. The presence of condensed tannins as the main phenolic components of PNSE was confirmed by chemical degradation methods.

## Experimental Section

### Materials

PNSE was obtained as previously described.^24^ Briefly, 1 g of PNS was extracted at room temperature with 2 × 10 mL of ethanol/water (6:4 v/v) for 30 min. 4042D PLA (94% l-lactic acid) was from NatureWorks LLC. Unstabilized linear low-density DJM1826 PE (2.5 g × 10 min^–1^ melt flow index) was purchased from Versalis. Anthocyanins were extracted from red wine pomace, black currant, and blackberry juice by adsorption on XAD7 HP (Sigma-Aldrich) and further purification on membrane adsorber Sartobind S IEX 150 mL (Sartorius Stedim Biotech).^33^ Red Delicious apples and bilberry juice (>40% fruit content) were purchased from local shops. All reagents and solvents were of analytical grade (Sigma-Aldrich).

### Structural Characterization of PNSE

#### Electron Paramagnetic Resonance (EPR) Analysis

EPR measurements were performed following an experimental procedure recently set up.^34,35^ Samples were measured using an X-band (9 GHz) Bruker Elexys E-500 spectrometer equipped with a superhigh sensitivity probe head. PNSE was introduced in a flame-sealed glass capillary coaxially inserted in a standard 4 mm quartz sample tube. Measurements were carried out at room temperature, with the following settings: sweep width, 140 G; resolution, 1024 points; modulation frequency, 100 kHz; modulation amplitude, 1.0 G, and receiver gain, 60 dB. The amplitude of the field modulation was preventively checked to be low enough to avoid detectable signal overmodulation. A microwave power of ∼0.6 mW was used to avoid microwave saturation of the resonance absorption curve. To improve the signal-to-noise ratio, 16 scans were accumulated. As concerning power saturation experiments, the microwave intensity was gradually increased from 0.004 to 127 mW. The *g* value and the spin density were determined using Mn^2+^-doped MgO as an internal standard.^35^ Spin density values must be considered as order of magnitude estimates, since sample hydration was not controlled.

#### Phloroglucinolysis

Phloroglucinolysis was conducted following a reported protocol^36^ with slight modifications. Briefly, 5 mg of PNSE was dissolved in 1 mL of methanol. One aliquot was used as the control to assess the content of free flavanols. Another aliquot of 100 μL was evaporated to dryness under nitrogen. Subsequently, 100 μL of the reaction solution containing ascorbic acid (10 mg/mL) and phloroglucinol (50 mg/mL) in 0.1 M methanolic HCl was added. After shaking for 20 min in a water bath at 50 °C, the reaction was quenched by adding 500 μL of aqueous sodium acetate (40 mM); after that, the mixture was filtered and analyzed by UHPLC-ESI-MS. A Waters Acquity i-Class instrument equipped with a binary pump, an autosampler (cooled to 10 °C, injecting 5 μL, a column oven (40 °C), and a diode-array detector was used. The column was a Nucleoshell phenyl-hexyl (2.0 mm × 150 mm, 2.7 μm, Macherey-Nagel), equipped with a security guard cartridge of the same material (2.0 mm × 5 mm, 1.8 μm). Eluant A was 0.1% formic acid in water, and eluant B was 0.1% formic acid in acetonitrile. A gradient elution program at a flow rate of 0.4 mL/min was used as follows: 1% B, 0–3 min; from 1 to 20% B, 3–22 min; from 20 to 100% B, 22–23 min; 100% B, 23–25 min. The mass spectrometer was a LTQ-XL ion trap system (Thermo Fisher Scientific) operating under the following detection conditions: sheath gas (N_2_), 52 arbitrary units; aux gas (N_2_), 1 unit; sweep gas (N_2_), 1 unit; ion spray voltage, 5 kV; capillary temperature, 300 °C; capillary voltage, 35 V; collision energy, 35 V.

The mean degree of polymerization (mDP) was calculated according to eq 1
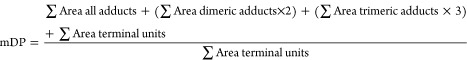
1

#### Thiolysis.^37^

Here, 8 mg of PNSE was treated with 2 mL of methanol, 20 μL of 37% HCl, and 50 μL of benzyl mercaptan (BM) at 40 °C under stirring. After 1 h, the mixture was diluted in 5 mL of methanol/water 1:1 v/v and directly analyzed by HPLC and LC-MS. HPLC analysis was run on an instrument equipped with an Agilent G1314A UV–vis detector, using a Phenomenex sphereclone ODS column (250 mm × 4.60 mm, 5 μm) at a flow rate of 1.0 mL/min; a gradient elution was performed with 0.1% formic acid (solvent A)/methanol (solvent B) as follows: 5% B, 0–10 min; from 5 to 80% B, 10–45 min; detection wavelength was 254 nm. LC-MS analyses were performed in a positive ionization mode on an Agilent LC-MS ESI-TOF 1260/6230DA instrument with the following parameters: nebulizer pressure, 35 psig; drying gas (nitrogen), 5 L/min; 325 °C; capillary voltage, 3500 V; fragmentor voltage, 175 V. An Eclipse Plus C18 column (150 mm × 4.6 mm, 5 μm) was used with the same mobile phase as above, at a flow rate of 0.4 mL/min.

#### Alkali Fusion.^38^

Here, 100 mg of KOH, 100 mg of NaOH, and 2 mg of Na_2_S_2_O_4_ were melted in a Pyrex tube at 240 °C. PNSE (20 mg) was then added, and the mixture was kept at 240 °C for a further 10 min. After cooling to room temperature, a 1% sodium dithionite solution (10 mL) was added. The resulting mixture was taken to pH 3 with acetic acid and then extracted with 3 × 15 mL of ethyl acetate. The organic layers were anhydrified with sodium sulfate and taken to dryness. The residue was dissolved up in methanol and analyzed by HPLC and LC-MS under the conditions described for thiolysis.

### Antioxidant Properties of PNSE

#### 2,2-Diphenyl-1-picrylhydrazyl (DPPH) Assay.^39^

Here, 20 μL of a 0.1–1 mg/mL PNSE solution in dimethyl sulfoxide (DMSO) were added to a 0.2 mM ethanolic solution of DPPH (2 mL), and the mixture was taken under stirring at room temperature. After 10 min, the absorbance at 515 nm was measured using an Agilent Hewlett Packard 8453 UV–vis spectrophotometer. Trolox was used as reference antioxidant. Each experiment was run in triplicate.

#### Ferric Reducing/Antioxidant Power (FRAP) Assay.^40^

Here, 20 μL of a 0.05–0.5 mg/mL PNSE solution in DMSO were added to a solution containing 1.7 mM FeCl_3_ and 0.83 mM 2,4,6-tris(2-pirydyl)-*s*-triazine in a 0.3 M acetate buffer (pH 3.6) (2 mL). The resulting mixture was vigorously stirred at room temperature, and the reduction of Fe^3+^ to Fe^2+^ was evaluated after 10 min by measurement of the 593 nm absorbance. An Agilent Hewlett Packard 8453 spectrophotometer was used. Each experiment was run in triplicate. Results were expressed as Trolox equivalents.

### Cell Viability Assays

Cytotoxic effects on human hepatocarcinoma (HepG2) cells were assessed using the 3-(4,5-dimethyl-2-thiazolyl)-2,5-diphenyl-2H-tetrazolium bromide (MTT) dye. HepG2 cells were obtained from the American Type Culture Collection (ATCC, Manassas, VA, USA) and grown at 37 °C in a humidified incubator containing 5% CO_2_ in Dulbecco’s modified Eagle’s medium (DMEM) supplemented with 10% fetal bovine serum, 4 mM glutamine, 400 U/mL penicillin, and 0.1 mg/mL streptomycin.

Cells were plated on 96-well plates at a density of 5 × 10^3^ per well in 100 μL of medium and incubated at 37 °C with 5% CO_2_. The medium was then replaced with 100 μL of fresh media containing PNSE at 5–50 μg/mL, and cells were incubated at 37 °C with 5% CO_2_. After 24–72 h incubation at 37 °C, the medium containing PNSE was withdrawn, and fresh medium (100 μL) containing 10% MTT was added to each well. After that, cells were incubated in darkness at 37 °C for 4 h. Cell survival was reported as the relative absorbance, with respect to control, of blue formazan measured at 570 nm with a Synergy Multi Plate Reader. Cell viability was reported as mean ± SD percentage.

### Preparation and Characterization of PE and PLA Films Containing PNSE

#### Film Preparation and Characterization by Scanning Electron Microscopy (SEM)

Extruded PE and PLA films were prepared as previously described.^24^ Briefly, PNSE was mixed with PE or PLA powder at 1%, 2%, or 3% w/w (grams of PNSE per grams of polymer). Then, the powder mixture was extruded with a flat die single-screw extruder, and films with an average thickness of 70 ± 10 μm were obtained by calendering. Higher concentrations of PNSE yielded visibly inhomogeneous films with poor mechanical properties.

Additionally, PLA films, neat and added with 3% or 10% w/w PNSE, were prepared through solvent casting. A suitable amount of PNSE was dissolved in 50 mL of a chloroform:methanol (8:2 v/v) solution by sonication over 30 min. Then, 2 g of PLA pellets was added, and the mixture was stirred for 120 min at 80 °C using a magnetic heater-stirrer. The resulting solution was transferred to a 12 cm-diameter Petri dish, and the solvent was allowed to evaporate slowly for 2 d to obtain films of 180 ± 10 μm thickness. The solvent-casting technique was not applicable to PE due to its extremely low solubility in most solvents.

Scanning electron microscopy (SEM) characterization of extruded and solvent-cast films was carried out using a FEI Quanta 200 FEG device. Before observation, the samples were coated with a Au–Pd alloy layer of 18.0 ± 0.2 nm, using a MED 020 Bal-Tec AG coater.

#### Antioxidant Properties of PE and PLA Films

##### DPPH Assay

Here, 2–10 PE or PLA (both extruded or solvent-cast) film sections of 1 cm^2^ total surface area (corresponding to ca. 20–100 mg of material) were introduced in a vial containing 20 mL of a 55 μM ethanolic solution of DPPH and allowed to sit up to 7 d at room temperature. Blank samples without the films were also prepared. The absorbance of each solution at 515 nm was periodically analyzed. Each experiment was performed in triplicate.

##### FRAP Assay

Here, 5 film sections of 1 cm^2^ total surface area (corresponding to ca. 50 mg of material) were introduced in a vial containing 20 mL of a FRAP solution prepared as described above and allowed to sit for 4 d at room temperature. The assay was performed on PLA (both extruded or solvent-cast) films containing or not PNSE. Blank samples without the films were also prepared. The absorbance of each solution at 593 nm was periodically analyzed. Each experiment was performed in triplicate. Results were expressed as Trolox equivalents. Experiments were run in triplicate.

#### Release of PNSE from PE and PLA Films in Assay Media

Five PE or PLA (both extruded or solvent-cast) film sections of 1 cm^2^ total surface area (corresponding to ca. 50 mg of material) were introduced in a vial containing 20 mL of ethanol or 0.3 M acetate buffer (pH 3.6) and allowed to sit for 4 d at room temperature. UV–vis spectra of each solution were periodically recorded. The amount of PNSE released was determined by means of a calibration curve obtained with 0.05–0.2 mg/mL PNSE solutions in the same solvents. Experiments were run in triplicate.

#### Film Mechanical Properties Characterization

Tensile tests were performed at 23 ± 2 °C and 45 ± 5% relative humidity (RH) on solvent-cast PLA films using a dynamometer (Instron 5564) equipped with a 1 kN load cell at a 5 mm min^–1^ clamp displacement rate. Before testing, the dumbbell-shaped specimens were conditioned at 25 °C and 50% RH for 48 h. At least 10 specimens were tested for each formulation. Mechanical characterization of extruded PE and PLA films has been already reported.^24^

#### FT-IR Analysis and Oxygen Transmission Rate (OTR) Measurement

FTIR-ATR spectra were recorded as an average of 16 scans in the range of 4000–450 cm^–1^ (resolution of 4 cm^–1^) on a PerkinElmer Spectrum 100 spectrometer equipped with a Universal ATR diamond crystal accessory. OTR was measured on solvent-cast PLA films by using a ExtraSolution PermeO_2_ instrument working in a gas/membrane/gas configuration, using a measuring surface of 50 mm at 50% RH, 23 ± 1 °C, and 1 atm of pressure difference (Δ*P*) across the membrane. The test was ended when the collected data reached an OTR accuracy within 0.5%. Oxygen permeability was obtained from OTR as follows (eq 2)
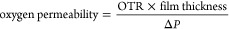
2

### Enzymatic Browning Inhibition Properties of PNSE

#### Mushroom Tyrosinase Inhibition Assay^41,42^

Here, 10 μL of a DMSO solution of PNSE was incubated at room temperature in 2 mL (0.02–0.25 mg/mL PNSE final concentration) of 50 mM phosphate buffer (pH 6.8) containing 20 U/mL of mushroom tyrosinase. After 10 min, 20 μL of a 100 mM l-DOPA or l-tyrosine solution in 0.6 M HCl was added (1 mM final concentration), and the absorbance at 475 nm was measured after an additional 10 min. In blank experiments, the reaction was run in the absence of PNSE. When required, the assay was carried out as described above, but l-DOPA was added to the reaction mixture soon after the addition of PNSE (0.06 mg/mL).

#### Apple Smoothie Browning Inhibition Assay

Red Delicious apples were rapidly cut in small pieces after removing the peel, and ca. 50 g were finely blended with a domestic mixer in the presence of a 0.085% PNSE solution (prepared by dissolving 100 mg of PNSE in 15 mL of DMSO followed by addition of 100 mL of double-distilled water) and transferred on a watch glass. Blank smoothie samples were prepared in the absence of PNSE. Changes in color were periodically analyzed with a Chroma Meter CR-400/410 (Konica Minolta) colorimeter (three different measurements were taken during the same experiments, and three different experiments were performed). The browning index (BI) was calculated as follows (eq 3)^43^
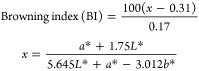
3

Kojic acid and ascorbic acid were used as reference compounds in parallel experiments.

In other experiments, smoothies prepared by blending 50 g of apple pulp in 100 mL of double-distilled water were rapidly transferred on a watch glass and immediately covered with solvent-cast PLA films, neat or containing PNSE. The changes in color were periodically analyzed with a colorimeter as above. Uncovered smoothies were used as blank samples.

### Anthocyanin Stabilization Properties of PNSE

#### Anthocyanin Stabilization Assay

The assay was performed as described.^44^ Briefly, to 5 mL of a 0.1 mg/mL red wine anthocyanin solution in a 0.3 M acetate buffer (pH 3.6), 40–250 μL of 1 mg/mL PNSE solution in DMSO was added (corresponding to 0.008–0.05 mg/mL final concentration of PNSE in the anthocyanin solution). The mixtures were taken at 90 °C and periodically analyzed by measuring the absorbance at 521 nm at 1 h intervals. Control mixtures containing the same amount of DMSO (without extracts) and unheated samples were analyzed in the same way. In other experiments, anthocyanins were incubated under the same conditions with 250 μL of a 1 mg/mL DMSO solution of chlorogenic acid or ferulic acid as reference compounds (0.05 mg/mL final concentration).

In another series of experiments, PNSE (2–10 mg) was added to 5 mL of bilberry juice, and the mixtures were taken at 90 °C and periodically analyzed as described above, following a 1:40 v/v dilution in a 0.3 M acetate buffer (pH 3.6).

Three different measurements were taken during the same experiments, and three different experiments were performed.

#### Storage Stability of Spray-Dried Anthocyanins

The spray-drying procedure followed a previously published method,^33^ using a Büchi B-290 lab-scale drier. Feed solutions contained 10 g/L of anthocyanins from blackberry or black currant juice and either 50 g/L of maltodextrin 19 (DE-value 18–20) for the control or 10 g/L of PNSE and 40 g/L of maltodextrin. All components were solubilized in ultrapure water (100 mL), which was taken in an ultrasound bath for 20 min. The solution was then filtered using a 595 Whatman paper filter (GE Healthcare). The following parameters were adopted: inlet temperature, 150 C; air-flow rate, 470 L/h; and aspirator set at 100% (approximately 35 m^3^/h). The resulting outlet temperature was kept between 96 and 99 °C by slightly adjusting the flow rate (approximately 5 mL/min). The storage stability of the resulting powders was determined by analysis of anthocyanin degradation using UHPLC-DAD.^33^ The samples were stored under UV light with or without exposure to oxygen at 35 °C for 10 weeks. Quantification of anthocyanins and calculation of half-lives (*t*_1/2_) were performed as previously reported.^33^ Samples were dissolved in water/acetonitrile/formic acid (80/15/5 v/v/v) at a concentration of 0.5 mg/mL. The CIELab color metrics were obtained as previosusly reported.^33^ The samples were diluted to a 1:2 ratio with water/acetonitrile/formic acid (80:15:5 v/v/v) after that absorption spectra were recorded with a Jasco V-730 spectrophotometer (Pfungstadt, Germany). The color parameters Chroma *C* and hue *h* and the resulting color difference Δ*E* were calculated as described previously.^33^

### Statistical Analysis

Data were processed by one-way analysis of variance using OriginPro 8.5. Significant differences among the means were assessed using Tukey’s test (*P* < 0.05).

## Results and Discussion

### Structural Characterization of PNSE

**Figure 1:**
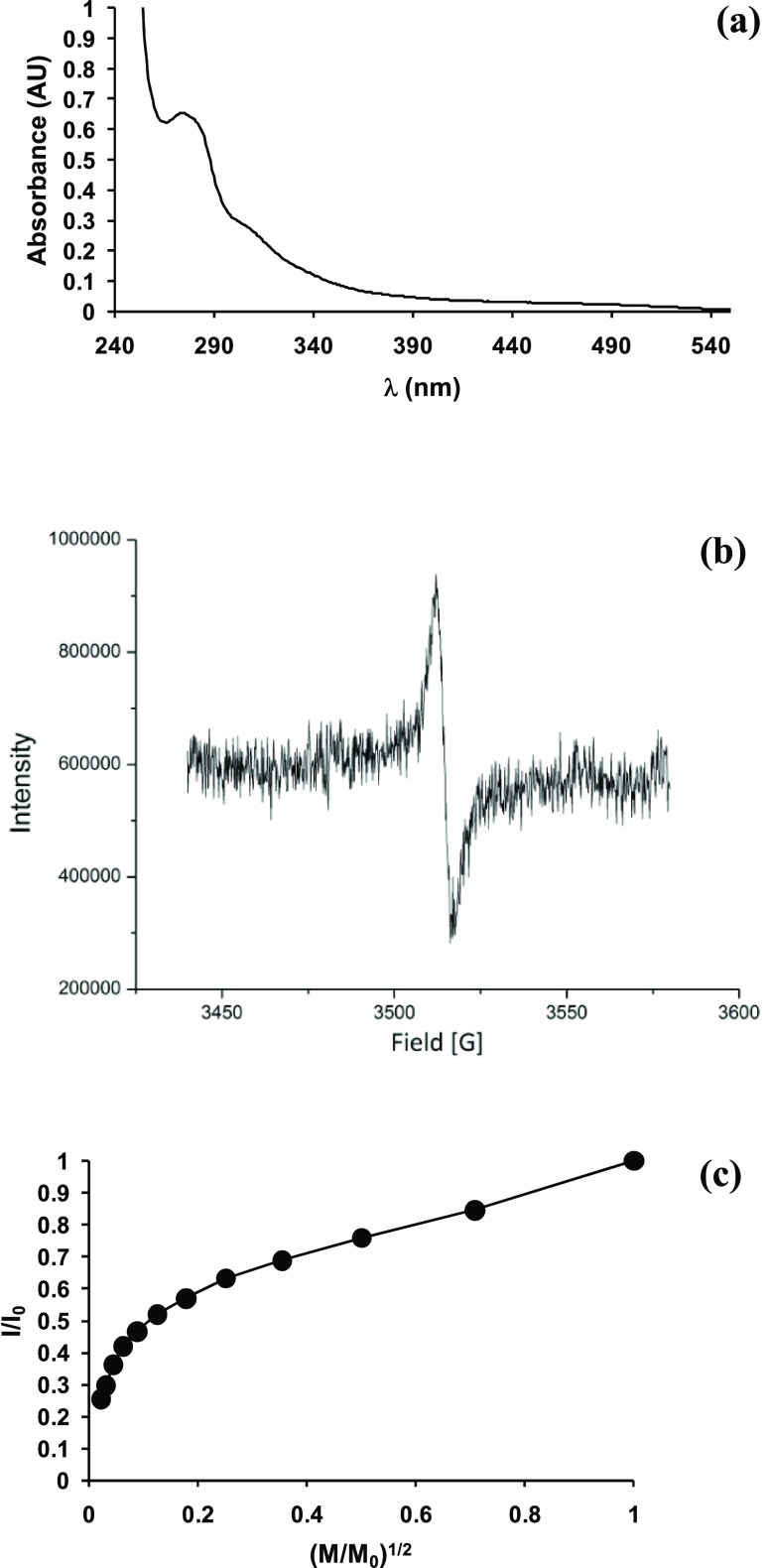
(a) UV–vis spectrum of a 0.2 mg/mL PNSE methanolic solution, (b) EPR spectrum of PNSE, and (c) EPR power saturation profile of PNSE.

HPLC analysis of PNSE dissolved in DMSO and diluted in methanol showed very low amounts of chromatographically defined compounds. A great chemical heterogeneity was apparent also from ^1^H NMR analysis, showing very broad signals in the aromatic region (not shown), whereas the UV–vis spectrum featured an absorption maximum at 278 nm, with a shoulder at around 300 nm (Figure 1a), indicative of the presence of phenolic moieties. EPR spectroscopy revealed a signal typical of a phenolic polymer (Figure 1b), characterized by a g value of 2.0035 ± 0.0002 due to carbon-centered radicals, a ΔB value of 5.1 ± 0.2 G, and a spin density of 5.4 ± 0.5 × 10^14^ spin/g. On the basis of the high (ca. 75%) percentage of Lorentian line shape, this relatively large ΔB value would be indicative of a low extent of π-electron conjugation across the phenolic polymer moieties.^35^ The observed slope change in the monotonously increasing trend of the normalized power saturation profile (Figure 1c) suggested that the paramagnetic centers in PNSE had a high extent of molecular heterogeneity.^35,45^

These results are in line with literature data reporting condensed tannins as the main phenolic components of PNS extracts.^24,46−51^ These are polymeric compounds constituted of terminal and multiple extension moieties related to flavan-3-ols linked by carbon–carbon bonds, which are commonly analyzed by chemical degradation treatments based on acid-catalyzed depolymerization in the presence of a strong nucleophile, followed by HPLC separation and quantification of individual polymer subunits. In this case, HPLC analysis of mixtures obtained by thiolysis, phloroglucinolysis, and alkali fusion revealed (epi)gallocatechin, (epi)catechin, and hydroxybenzoic acids as the main cleavage products (Table 1 and Figure S1). In particular, phloroglucinolysis experiments revealed a rather high mDP value (Table 1) and (epi)gallocatechin as the most abundant terminal and extension unit, suggestive of prodelphinidin-type tannins.

**Table 1:** Composition of Proanthocyanidins in PNSE Determined by Phloroglucinolysis Experiments^a^

Compound	Composition of terminal units (%)	Composition of extension units (%)
Catechin	33.4	6.0
Epicatechin	0	15.8
(Epi)gallocatechin	53.3	77.4
Epicatechin gallate	1.5	0.2
(Epi)gallocatechin gallate	3.1	0.3
(Epi)afzelechin	8.2	0.2
A-type dimers	0	0.1

a Calculated mean degree of polymerization (mDP) is 20.1.

### Antioxidant Properties of PNSE

Several articles have described PNS as a source of polyphenols using different extraction procedures, and these polyphenols, mainly condensed tannins, have been regarded as a viable alternative for food supplements and nutraceuticals as well as for the treatment of cancer and other pathologies, given their effective antioxidant properties.^46−52^ In this work, PNSE was obtained by treatment of nut shell with ethanol and water (6:4 v/v) under the conditions previously developed.^24^ The antioxidant properties of PNSE were investigated by two widely used assays, i.e., the DPPH assay, which determines the electron donor capacity by the spectrophotometric monitoring at 515 nm of a DPPH solution containing the sample,^39^ and the FRAP assay, in which the ability of the sample to reduce a Fe^3+^-tripyridyltriazine complex to a dark blue Fe^2+^ complex is measured by monitoring the absorbance at 593 nm.^40^ A EC_50_ value of 4.00 ± 0.01 μg/mL was determined in the DPPH assay, confirming a very high efficiency of PNSE when compared to the standard antioxidant Trolox, which under the same conditions exhibited a EC_50_ value of 6.00 ± 0.02 μg/mL. On the other hand, a value of only 0.183 ± 0.007 Trolox equivalents was measured in the FRAP assay, likely due to a lower PNSE solubility in the aqueous assay medium.

### Biocompatibility of PNSE

To assess the biocompatibility properties of PNSE, possible toxic effects on HepG2 cells were evaluated by a MTT reduction assay at three different times of incubation (24, 48, and 72 h). HepG2 cell line is widely used as a model system of the human liver to study the toxicity as well as the chemopreventive potential of different substances.^53^ The results are presented in Figure S2 and show that PNSE does not exert significant toxic effects over a time interval of 72 h at concentrations as high as 50 μg/mL, which can be considered physiological and realistic in the perspective of using PNSE as a food additive.^23^ These results well compare with literature data reporting no motor, gastric, or toxicological alterations in mice treated with PNS extracts.^48,54−57^

### Antioxidant Properties of PE and PLA Films Containing PNSE

In subsequent experiments, the possible use of PNSE as a functional additive for active packaging was evaluated. To this aim, PLA or PE films containing PNSE at 1%, 2%, or 3% w/w were prepared by extrusion as described in a previous work^24^ and tested in the DPPH assays in comparison with control films not containing PNSE. The incorporation of the extract introduced an antioxidant functionality in a dose-dependent manner in both polymers. This effect was particularly marked in the case of PLA films, leading to more than 80% DPPH reduction after 1 week with a 3% w/w loading of PNSE (Figure S3).

**Figure 2:**
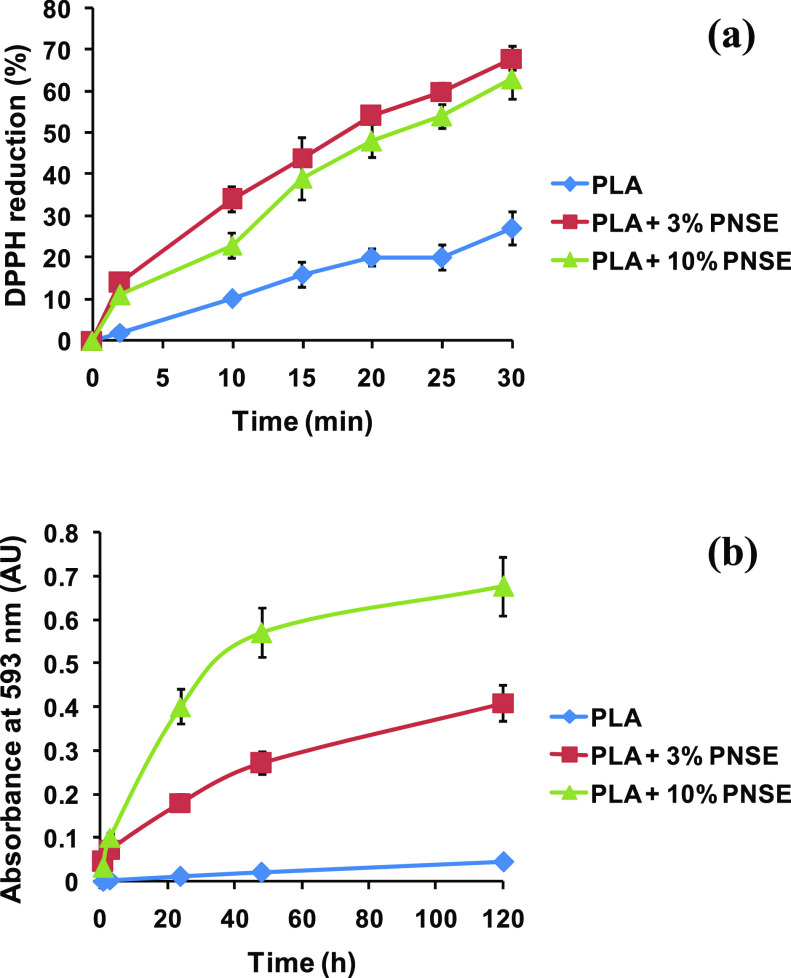
(a) DPPH-reducing activity of solvent-cast PLA films containing PNSE. (b) Fe^3+^-reducing activity of solvent-cast PLA films containing PNSE. Mean ± SD values of three experiments are reported.

Remarkably higher antioxidant properties were observed in the DPPH assay for solvent-cast PLA films compared to those obtained by extrusion, since even at a dose as low as 1 mg/mL in the DPPH solution they were able to induce a 60% reduction after only 30 min (Figure 2a).

The solvent-casting technique was not applicable to PE due to its extremely low solubility in most solvents.

The differences in the antioxidant properties of the tested films may be ascribed both to the nature of the polymer and to the technique used to obtain the film. During extrusion, high temperature causes the polymer to melt, while the mechanical shear due to screw rotation allows for comminution of the additive and blending with the polymer matrix. The subsequent roll calendering process results in the formation of a thin film in which the finely dispersed additive particles are confined inside the bulk of the film.

**Figure 3:**
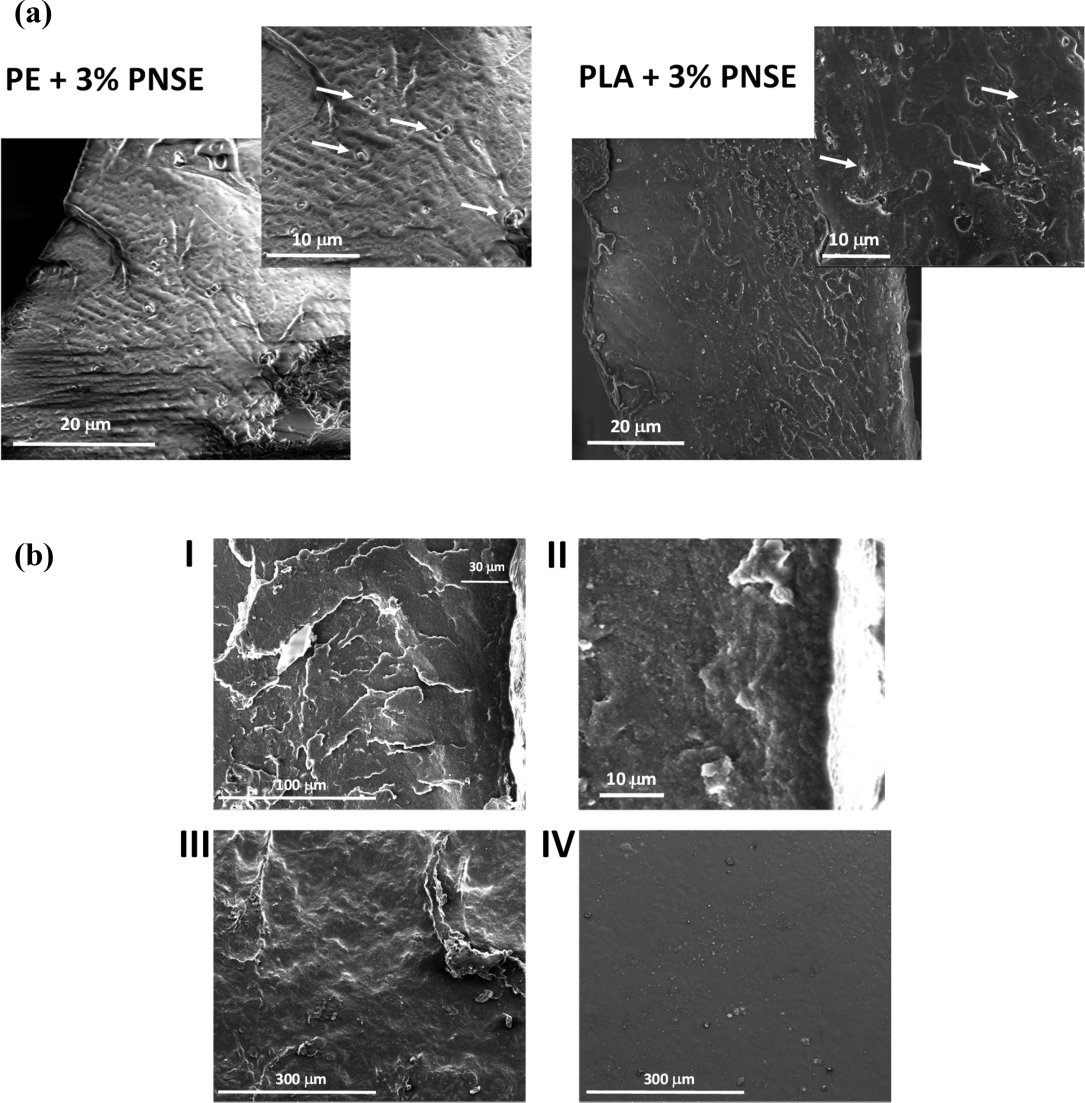
(a) SEM images of cross sections of extruded PE + 3% PNSE (left) and PLA + 3% PNSE (right) films; PNSE particles are indicated by arrows. (b) SEM images of cross section (I and II), top surface (PNSE-rich) (III), and bottom surface (PLA-rich) (IV) of solvent-cast PLA + 10% PNSE films.

Figure 3a shows the cross sections of extruded PE and PLA films containing PNSE, highlighting the presence of submicrometer-sized PNSE particles (indicated by arrows) well distributed in PE, while some particle agglomerates can be observed in the case of PLA. Efficient particle embedding entails that migration of PNSE from the bulk of the film into the solution is hampered, also accounting for the poor antioxidant properties measured in the DPPH assay.^58,59^ This applies in particular to PE, which is a hydrophobic polymer with low wettability in polar solvents. Indeed, release experiments showed no migration of PNSE in the DPPH assay medium from the extruded films.

As far as solvent-cast PLA films are concerned, the absence of mechanical shear and the slow solvent evaporation cause the polymer to settle on the bottom of the Petri dish, while the extract remains on the top layer, leading eventually to a coating layer of about 30 μm on the film surface (Figure 3b). Therefore, higher diffusion of PNSE from solvent-cast PLA films is expected, explaining the higher antioxidant properties determined in the DPPH assay. Actually, parallel experiments indicated the release of almost all of PNSE from solvent-cast PLA films under the conditions of the DPPH assay after 24 h, following different kinetics depending on the percentage of incorporation of PNSE (Figure S4). In particular, a faster diffusion of PNSE was observed in the case of a lower loading concentration, likely as the result of a thinner and hence more permeable layer over the surface of the film.

The antioxidant properties of the PLA films were evaluated also in an aqueous medium by the FRAP assay (Figure 2b). Trolox equivalent values of 0.78 × 10^–3^ and 1.26 × 10^–3^ were exhibited by solvent-cast PLA films with a PNSE loading of 3% and 10% w/w, respectively, at 96 h. In contrast, extruded PLA films containing 3% w/w PNSE exhibited a reducing activity which was not so different from that of control extruded PLA films not containing PNSE (Figure S5). Notably, in this case, no significant migration was observed either for solvent-cast or extruded films, so it is unlikely that the differences found in the reducing properties could be ascribed to a release of PNSE in the assay medium.

On the basis of the results of the antioxidant assays, indicating a lower performance of extruded films compared to solvent-cast PLA films in view of their potential use in the food industry, only the latter were further investigated and characterized in the subsequent experiments.

### Characterization of Solvent-Cast PLA Films Containing PNSE

**Figure 4:**
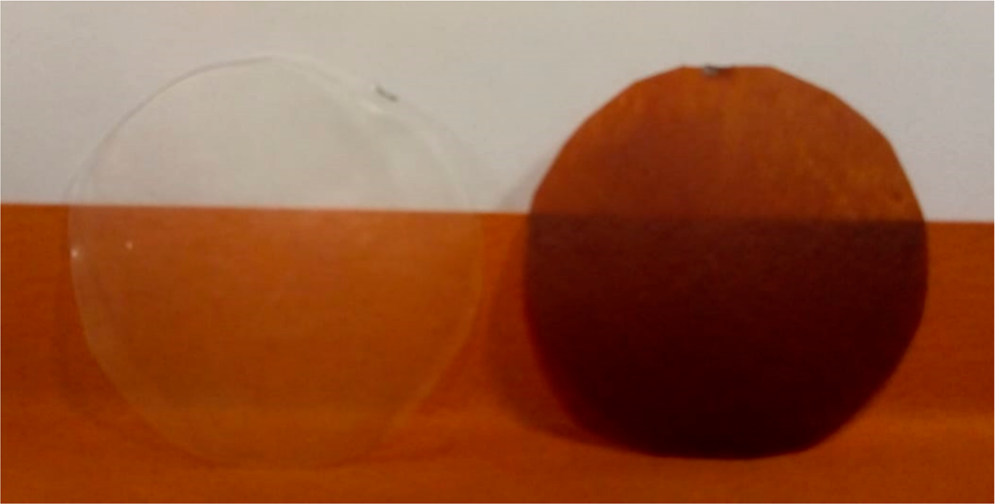
Digital photo of solvent-cast neat PLA film (left) and PLA film containing 10% w/w PNSE (right).

The PLA films containing PNSE showed a fairly homogeneous dark red coloration (Figure 4).

**Figure 5:**
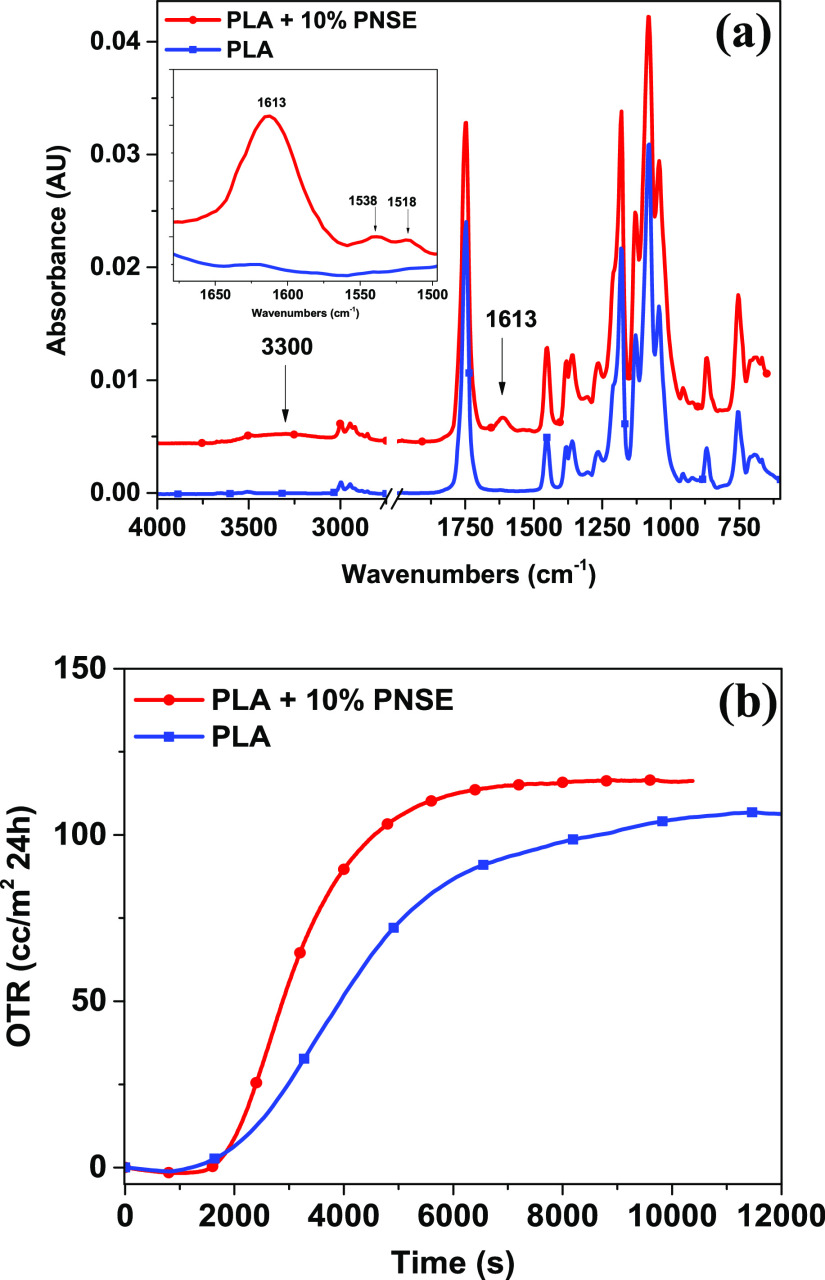
(a) FTIR-ATR spectra and (b) OTR curves of solvent-cast neat PLA films and PLA films containing 10% w/w PNSE.

The effect of the addition of PNSE on the structure of the solvent-cast PLA film was assessed by FTIR spectroscopy (Figure 5a and Figure S6). The main spectral changes due to the presence of phenolic moieties were the appearance of absorption at 3300 cm^–1^ (phenol O–H stretching), 1613 cm^–1^ (aromatic C=C), and 1538 and 1515 cm^–1^ (in-plane bending of phenyl C–H bonds).^17,24^ Notably, no changes were observed in the absorption frequency of the PLA carbonyl groups (1750 cm^–1^), indicating the absence of significant interactions between the polymer and the additive.

The mechanical properties of the solvent-cast films were measured by tensile tests. Strain and stress at break and elastic modulus values of PLA and PLA + 10% PNSE are shown in Table 2.

**Table 2:** Mechanical Properties of Solvent Cast PLA Films

	Strain at break^a^ (%)	Modulus^a^ (MPa)	Stress at break^b^ (MPa)
PLA	41 ± 20	1414 ± 411	25 ± 2*
PLA + 10% PNSE	32 ± 18	1454 ± 220	20 ± 2*

a Average of three determinations ± standard deviation.

b Starred values in the same column are significantly different (*P* < 0.05).

**Figure 6:**
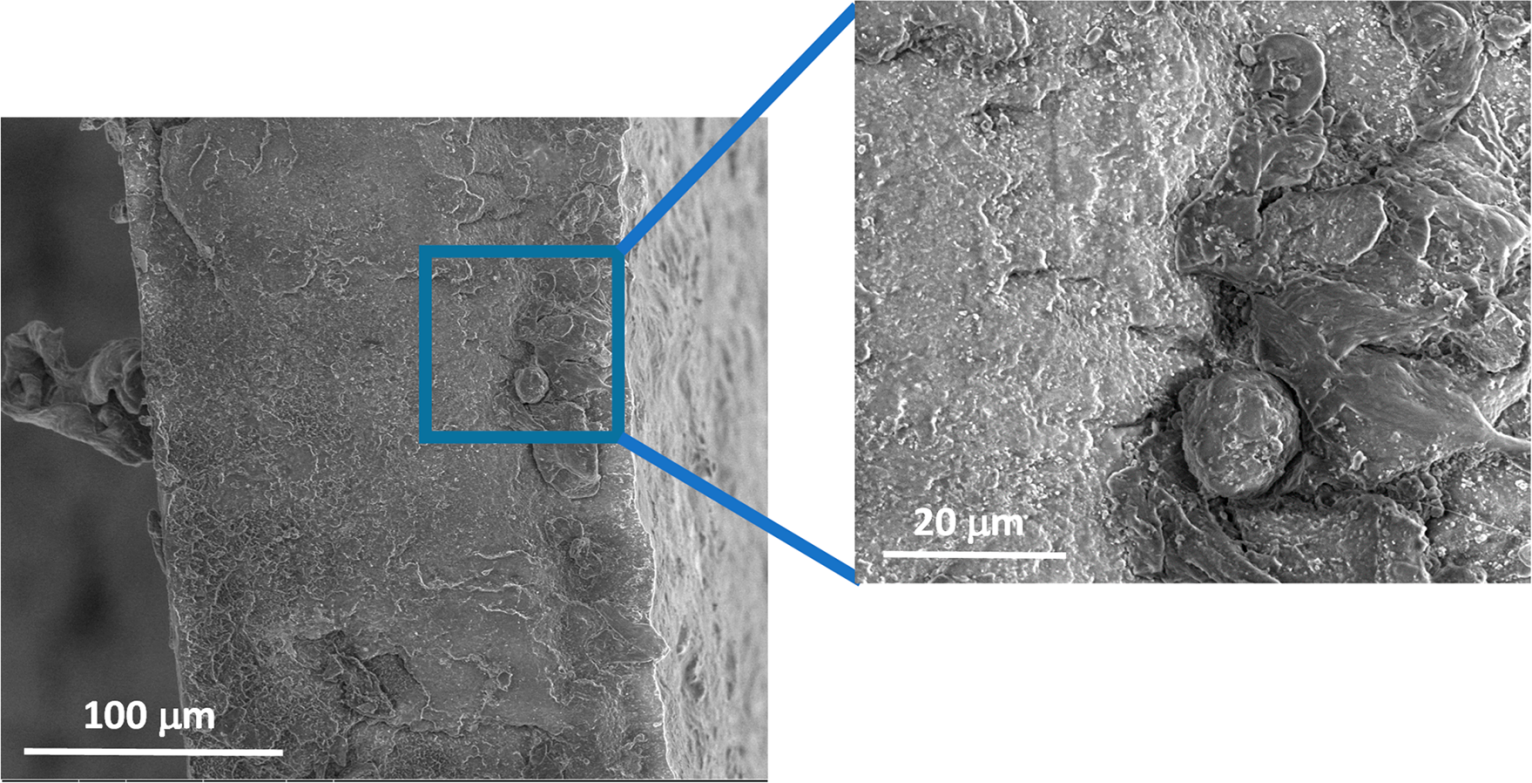
SEM images of cross section of solvent-cast PLA + 10% PNSE film, highlighting the presence of PNSE aggregates.

PNSE slightly, although not significantly, affected the strain at break value of PLA, which decreased from 41% to 32%. This apparent drop in the mechanical properties can be attributed to the formation of PNSE aggregates during solvent evaporation (Figure 6). These defects are able to initiate cracks which accelerate the sample failure.^24^ On the contrary, the addition of PNSE did not affect the mechanical performance of solvent-cast PLA films in terms of modulus, considering that a slight increase in modulus is generally expected in the presence of natural additives or fillers.^17,24,60^ In other experiments, the oxygen permeability of the solvent-cast films was determined based on the experimental OTR values (Figure 5b). Values of 8.33 ± 0.46 × 10^–16^ and 8.26 ± 0.50 × 10^–16^ g m/m^2^ s Pa were found for solvent-cast neat PLA films and PLA films containing 10% w/w PNSE, respectively, indicating that PNSE did not significantly affect the oxygen permeability property of the film. This could be the result of the preferential localization of PNSE on the film surface (Figure 3b) entailing that the film bulk is essentially made of almost pure PLA, with a negligible influence on the gas permeability. Further, the recorded values were in good agreement with literature data^61,62^ and compatible with food packaging applications.^63,64^

### Enzymatic Browning Inhibition Properties of PNSE

To further expand the possible applications of PNSE in the food sector, in subsequent experiments, the inhibitory properties of PNSE toward mushroom tyrosinase were investigated. Tyrosinase belongs to the enzyme class of polyphenol oxidases, playing a critical role in the oxidative deterioration of food products, particularly fruit and vegetables. It is a copper-containing enzyme which catalyzes the hydroxylation of monophenols to *o*-diphenols (monophenolase or cresolase activity) and the oxidation of diphenols to quinones (diphenolase or catecholase activity). Molecular oxygen acts as the electron acceptor in both reactions. Quinones are highly reactive species and can undergo nonenzymatic polymerization reactions ultimately leading to brown pigments. Enzymatic browning in spoiled fruits and vegetables during postharvest handling and processing represents an important problem in the food industry and for the consumers, because it leads to undesirable, potentially toxic products. Natural tyrosinase inhibitors have therefore attracted strong interest in the food sector to control the rate of this process.^65^ Tyrosinase inhibition is an increasingly important target also for the cosmetic industry, as this enzyme catalyzes melanogenesis key steps in humans, that is hydroxylation and oxidation of l-tyrosine to dopaquinone; overproduction of melanin is associated with several pigmentary disorders, whose major medical and aesthetical consequences have led to the quest for new nontoxic depigmenting agents.^66,67^

The ability of PNSE to inhibit mushroom tyrosinase was preliminarily investigated using l-dopa or l-tyrosine as the substrate. The assays is based on the detection of dopachrome formation (λ_max_ 475 nm) following oxidative cyclization of dopaquinone resulting from the tyrosinase-induced oxidation of the substrate, with or without inhibitor.^42^ As reported in Figure S7, quite low IC_50_ values were observed for both cresolase and particularly catecholase activity of the enzyme. These values were comparable to those previously reported for condensed tannins from *Vigna angulariz* seeds,^68^ cherimoya pericarp,^69^ fig leaves,^70^ and longan bark.^71^ Under the same conditions, the well-established depigmenting agent kojic acid^66^ exhibited a IC_50_ value for the catecholase activity of ca. 50 μM, that is, 7 μg/mL.

**Figure 7:**
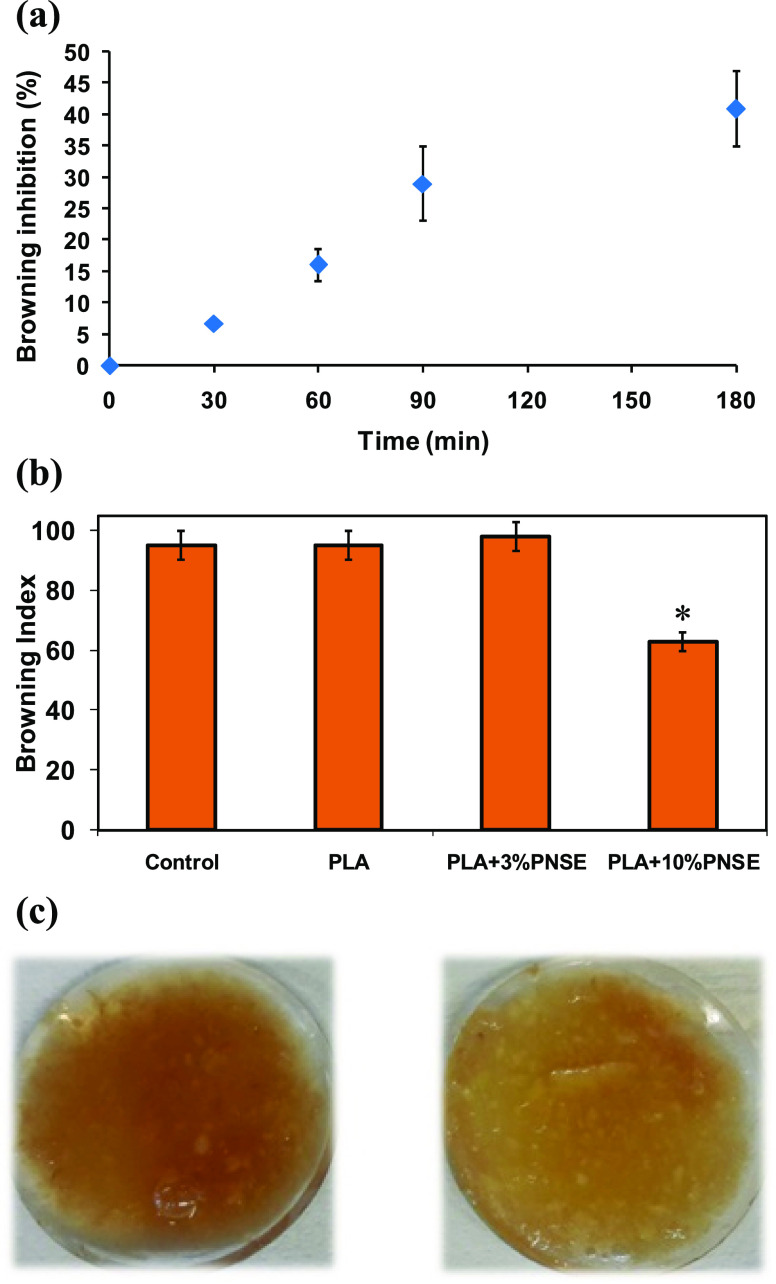
(a) Effect of PNSE on apple smoothie enzymatic browning at varying times. Mean ± SD values of three experiments are reported (three different measurements were taken during each experiment). (b) Browning index calculated after 3 h for apple smoothies covered with solvent-cast PLA films. Mean ± SD values of three experiments are reported (three different measurements were taken during each experiment). *P < 0.05 compared to control. (c) Apple smoothies covered with solvent-cast neat PLA film (left) and PLA film containing 10% w/w PNSE (right).

On the basis of these encouraging results, the enzymatic browning inhibition properties of PNSE were then evaluated in apple smoothies. Red Delicious apples were chosen for this study because of their high susceptibility to browning.^72^ In particular, apples were finely grinded with a hand blender in the presence or absence of a 0.1% w/v PNSE solution, and the changes in color development were periodically analyzed with a colorimeter. A significant inhibition of browning was observed in the presence of PNSE, peaking to a value of about 40% at 3 h (Figure 7a).

In parallel experiments, kojic acid and ascorbic acid were used as reference compounds. As mentioned before, the first is a well-known tyrosinase inhibitor acting through chelation of copper at the active site,^66^ whereas ascorbic acid is an antioxidant able to reduce quinonoid species thus preventing their polymerization;^73^ in addition, the lowering of the pH caused by ascorbic acid leads to a decrease of the enzymatic activity, which is maximal at pH 6–7. Under the same experimental conditions, kojic acid was more effective than PNSE, leading to a ca. 60% inhibition at 3 h. In contrast, ascorbic acid was able to induce an effect only in the first 2 h, after that no browning inhibition was observed.

### Enzymatic Browning Inhibition Properties of Solvent-Cast PLA Films Containing PNSE

To assess the ability of PNSE to delay enzymatic browning processes even when incorporated in PLA films, in further experiments, finely grinded apples were rapidly transferred on a watch glass and covered with the films, and the changes in color development were analyzed as described above. Figure 7b displays the browning index calculated after 3 h. Although PLA alone or with a loading of extract of 3% w/w did not exert any effect on enzymatic browning in the presence of 10% w/w PNSE a 30% inhibition was observed (Figure 7c). On the basis of the findings reported above, this effect cannot be ascribed to a change of the oxygen barrier properties of the films due to the presence of PNSE.

### Anthocyanin Stabilization Properties of PNSE

**Figure 8:**
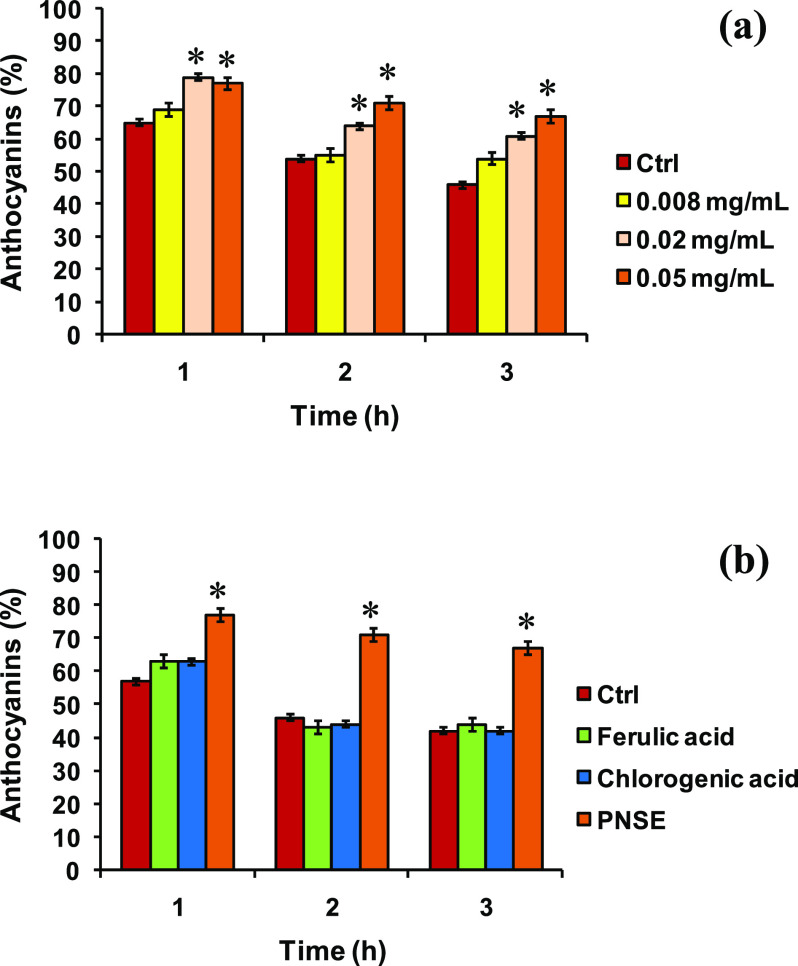
(a) Effect of PNSE at different doses and (b) comparison with ferulic acid and chlorogenic acid (all 0.05 mg/mL) on a red wine–anthocyanin solution stability at 90 °C. Mean ± SD values of three experiments are reported (three different measurements were taken during each experiment). *P < 0.05 compared to control.

In a final set of experiments, the effect of PNSE on anthocyanin stability was determined. Anthocyanins are natural pigments responsible for the blue, purple, and red colors of many fruits and vegetables. They are commonly used as food additives not only to impart specific color but also in light of their health-beneficial properties, including antioxidant, anti-inflammatory, antidiabetic, and anticancer activities.^74−76^ However, anthocyanins isolated from natural sources suffer from extensive degradation with consequent color loss, depending on several factors including light, pH, and temperature.^77−79^ For these reasons, several studies have been recently devoted to the evaluation of the effects of natural additives as stabilizers of anthocyanin color.^80^ Organic acids, metal ions, phenolic compounds, and aromatic amino acids like tryptophan have been found to inhibit anthocyanin degradation in model systems.^81−83^ In particular, phenolic compounds such as hydroxycinnamic acids and tannins are able to form noncovalent complexes with anthocyanins through π-stacking interactions, and this “copigmentation” phenomenon prevents water addition to the flavylium ion causing its degradation, with an overall effect of color stabilization.^84^ Copigmentation has also been shown to improve color intensity.^85^ The use of copigments to increase stability of anthocyanins in red wine or fruit- and berry-derived food and beverages is now widely documented. Moreover, enhanced anthocyanin storage and heat stability has been reported as a consequence of copigmentation, too.^86,87^ Hence, a further objective of this study was the evaluation of the effect of PNSE on red wine anthocyanin stability at 90 °C, under literature-reported conditions.^44^ When added to a 0.1 mg/mL anthocyanin solution (PNSE final concentration 0.008–0.05 mg/mL), no significant hyperchromic or bathochromic effect was observed, ruling out the occurrence of any copigmentation effect. However, PNSE was found to improve the heat stability of the pigments even at a concentration as low as 0.02 mg/mL (Figure 8a). Notably, at the same concentrations, no stabilizing effect was observed with the well- known anthocyanin copigments ferulic acid and chlorogenic acid (Figure 8b).^88,89^ Moreover, PNSE was found to be effective at lower concentrations compared to other polyphenol-rich extracts.^87^

To determine the efficiency of PNSE in a more complex food system, the anthocyanin-stabilizing properties of the extract were then evaluated in a commercial bilberry juice. As reported in Figure S8, a dose-dependent effect was observed also in this case, with an almost 50% lower decrease in anthocyanin content with respect to the control samples in the case of a PNSE dose of 2 mg/mL.

Future studies will be aimed at providing deeper insights into the mechanisms involved in the anthocyanin stabilization induced by PNSE.

Application of anthocyanins as natural food colorants necessitates their stabilization not only in solutions but also as a free-flowing powder which could ensure safe and easy dosage during processing. To this aim, spray drying has been applied in numerous studies, as a mild method to dry anthocyanin extracts.^90^ Additional stabilization of the dried anthocyanins can be achieved by supplementing the feed solution with other phenolic compounds that act as copigments.^33^ In the present study, two different anthocyanin extracts from black currant and blackberry juice were spray dried with or without PNSE. Characterization of the powders obtained by spray drying are reported in Table 3. The lower total yields observed for the samples with PNSE might be explained by the low solubility of PNSE in the aqueous solution, and the resulting decreased stability of the feed solution emulsion.

**Table 3:** Characterization of Powders from Spray-Drying Experiments

	Total yield (%)	Residual moisture (%)	Anthocyanin content (%)	CieLab chroma	CieLab hue
Black currant (BC)	83.0	4.0	101.0^a^	44.5	2.8
Black currant and PNSE (BC + PNSE)	63.3	6.3	105.0^a^	46.4	4.3
Blackberry (BB)	80.3	5.7	78.4	49.7	8.9
Blackberry and PNSE (BB + PNSE)	63.1	6.5	66.4	45.0	8.4

a Yield might be overestimated due to quantification as cyanidin 3-glucoside equivalents.

The resulting powders were then stored under UV light in open or evacuated and sealed Petri dishes at 35 °C for 10 weeks. The anthocyanin profile of blackberry is dominated by cyanidin 3-glucoside,^91^ whereas in black currant delphinidin derivatives are most abundant.^92^ The stability of the black currant anthocyanins during spray drying was significantly higher compared to the blackberry anthocyanins (Table 3), which is surprising because delphinidin derivatives have been shown to be more heat sensitive than the respective cyanidin derivatives.^93,94^

**Figure 9:**
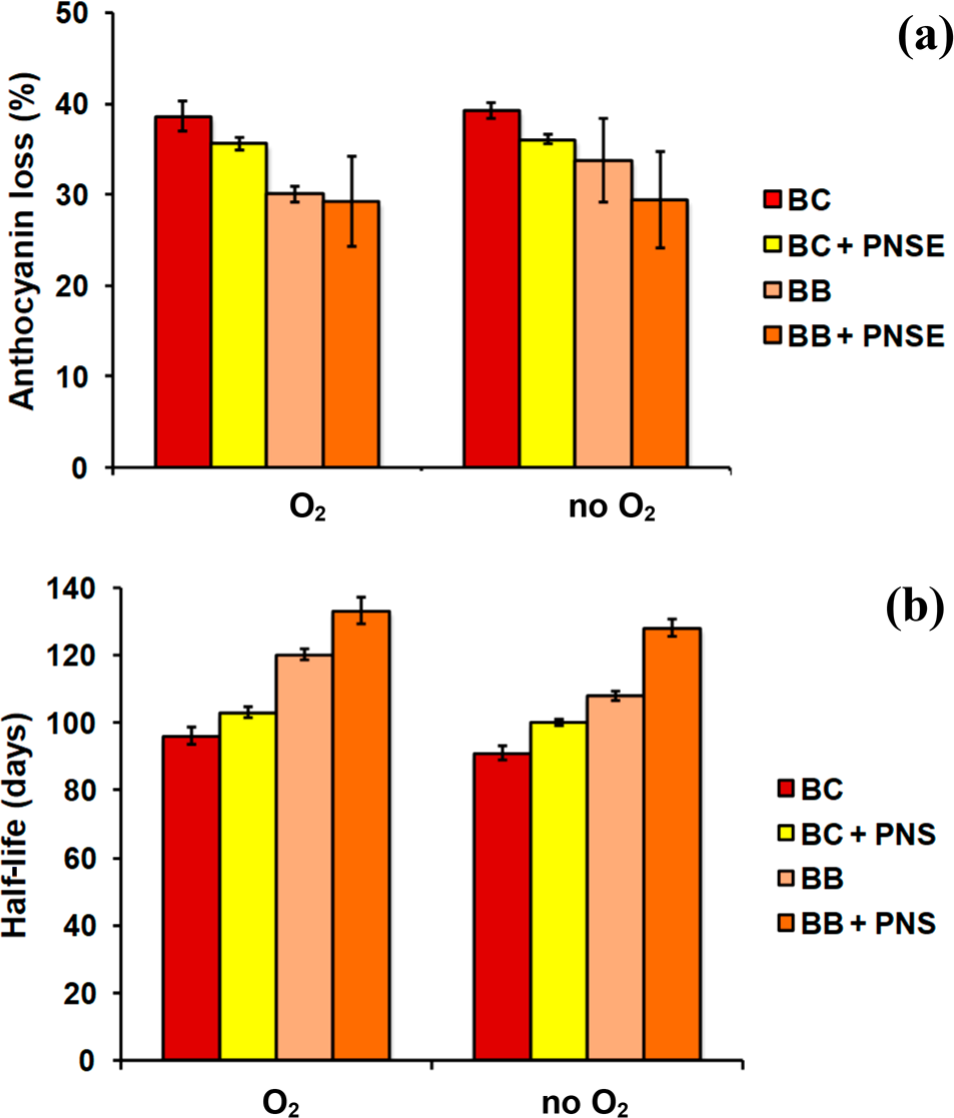
(a) Loss of spray-dried anthocyanins during storage and (b) calculated half-life of spray-dried anthocyanins after storage under UV light in open (O_2_) or sealed (no O_2_) Petri dishes. Mean ± SD values of three experiments are reported.

In the present study, a higher susceptibility of delphinidin derivatives was observed during storage, since the anthocyanins from blackberries were retained in higher concentrations after storage than the black currant anthocyanins (Figure 9). It was previously shown that addition of pigments could improve the stability of blackberry anthocyanins during dry storage.^33^ This could be confirmed in the present study, particularly when no oxygen was present.

## Conclusions

In conclusion, in this article, the possible exploitation of PNS as a low cost polyphenol source for use in active packaging and food preservation is reported. Apart from acting as an antioxidant, the hydroalcoholic extract of PNS, consisting mainly of condensed tannins, can efficiently delay enzymatic browning in fruit even when incorporated in solvent-cast PLA films. These films exhibited also good antioxidant properties both in organic and in aqueous solvents, therefore emerging as potential packaging materials for oxidative stabilization of foods. In particular, the low amounts of PNSE released from the films when taken in aqueous media, reaching concentrations that were shown to be of no toxicity for HepG2 cells, would suggest their applications to water-rich foods. On the other hand, the more substantial migration observed in organic solvents would warrant even higher oxidative stability in the case of low water content foodstuffs, such as oils, butters, and sauces. This is a result of considerable value in light of the literature data indicating very low toxicity of PNSE in animal models.

Preliminary experiments showed that PNSE is also able to stabilize anthocyanin pigments from thermal degradation in food, displaying higher efficiency compared to well-known copigments. Application of PNSE as a copigment during spray drying of anthocyanins showed promising results, which might be further enhanced by adjustment of the composition of the feed solution and optimization of the spray-drying parameters.

These results fulfill most of the 12 Principles of Green Chemistry,^95^ namely, (a) Less Hazardous Chemical Synthesis and Designing Safer Chemicals (pecan nut shell and PLA are of natural origin, and PNSE was found to be nontoxic in cellular assays), (b) Safer Solvents and Auxiliaries (only water and ethanol are needed to obtain PNSE, and only water was used to prepare the stabilized spray-dried food colorant powder), (c) Use of Renewable Feedstocks (PNSE is a food waste-derived product), and (d) Design for Degradation (PLA is a degradable polymer).

Although still preliminary for the purposes of a real application, these results, coupled with previous evaluations demonstrating the technical and economic feasibility of PNSE manufacturing,^24^ would make PNSE a highly convenient and competitive functional additive for the food industry.

## Supporting Information Available

Elution profiles of the chemical degradation mixtures of PNSE, data from cell viability experiments, DPPH-reducing properties of extruded films, release of PNSE from solvent-cast PLA films, Fe^3+^ reducing activity of extruded PLA films, close up view of the FTIR spectra of solvent-cast PLA films, data from mushroom tyrosinase inhibition assays, and effect of PNSE on anthocyanin stability in bilberry juice (PDF)
