# Supplementary material for: Pecan (Carya illinoinensis (Wagenh.) K. Koch) Nut Shell as an Accessible Polyphenol Source for Active Packaging and Food Colorant Stabilization
Source: ACS Sustain Chem Eng. 2020 Apr 13;8(17):6700–12. doi: 10.1021/acssuschemeng.0c00356 (PMC8016391; doi:10.1021/acssuschemeng.0c00356)
Supplement: Supplementary file 1 — sc0c00356_si_001.pdf [file sc0c00356_si_001.pdf]

Pecan (*Carya illinoinensis* (Wagenh.) K. Koch) Nut Shell as an Accessible Polyphenol Source for Active Packaging and Food Colorant Stabilization

Federica Moccia, Sarai Agustin-Salazar, Anna-Lisa Berg, Brunella Setaro, Raffaella Micillo, Elio Pizzo, Fabian Weber, Nohemi Gamez-Meza, Andreas Schieber, Pierfrancesco Cerruti,* Lucia Panzella,* and Alessandra Napolitano

*Corresponding author: cerruti@ipcb.cnr.it (PC)

*Corresponding author: panzella@unina.it (LP)

Table of contents

**Figure S1.** Elution profiles of the chemical degradation mixtures of PNSE.	Pag. S2
**Figure S2.** Effect of PNSE on HepG2 cell viability determined by MTT assay.	Pag. S3
**Figure S3.** DPPH reducing activity of extruded PE and PLA films.	Pag. S4
**Figure S4.** Release of PNSE from solvent-cast PLA films under the conditions of the DPPH assay.	Pag. S5
**Figure S5.** Fe^{3+} reducing activity of extruded PLA films.	Pag. S6
**Figure S6.** Close up view of the 4000-2500 cm^{-1} region of FTIR spectra of solvent-cast PLA and PLA + 10% PNSE films.	Pag. S7
**Figure S7.** Percentage of inhibition of mushroom tyrosinase activity *vs* PNSE concentration.	Pag. S8
**Figure S8.** Effect of PNSE on anthocyanin stability at 90 °C in a bilberry juice.	Pag. S9

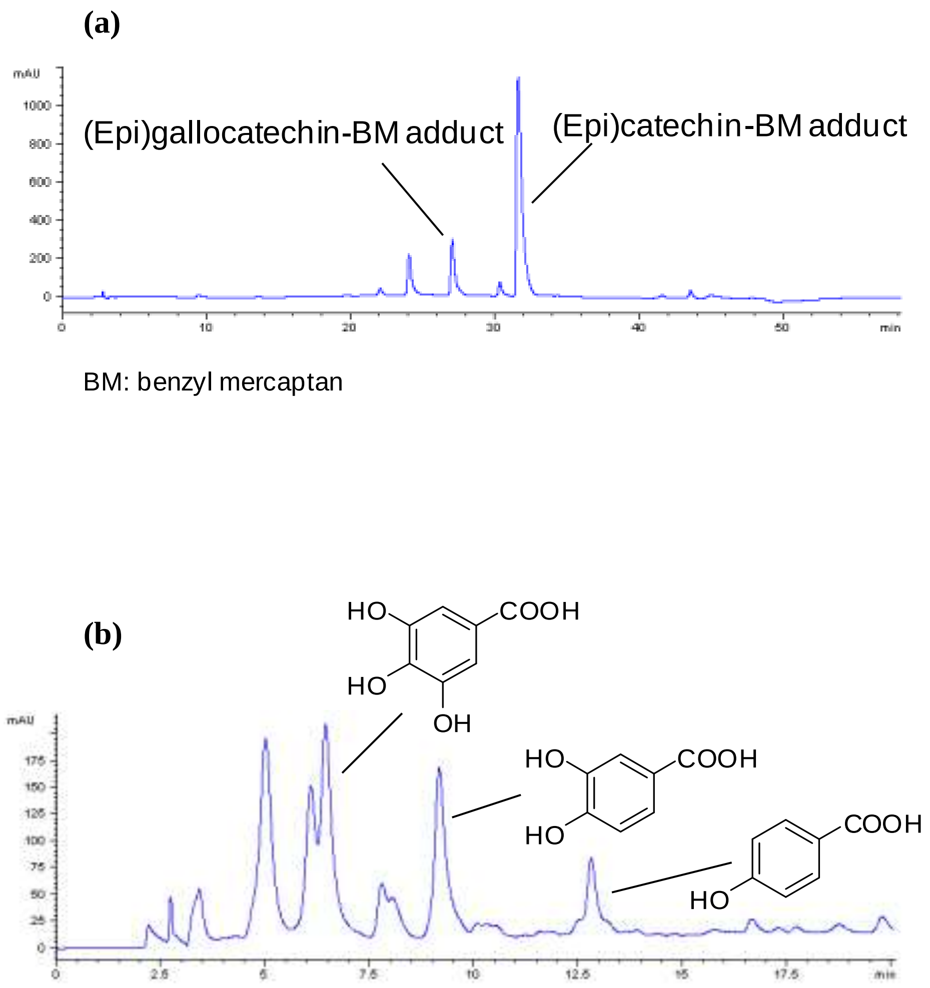

Figure S1. Elution profiles of the chemical degradation mixtures of PNSE. (a) Elution profile and identified compounds of the thiolysis mixture. (b) Elution profile and identified compounds of the alkali fusion mixture.

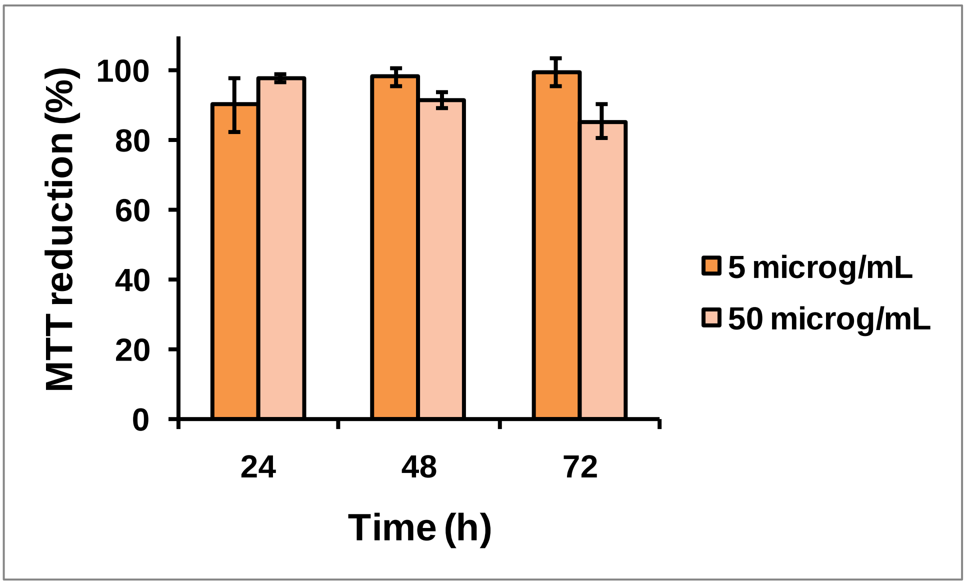

Figure S2. Effect of PNSE on HepG2 cell viability determined by MTT assay. Cells were cultured in DMEM medium and then subjected to treatment with different concentrations of PNSE for 24 h, 48 h and 72 h. Cell viability was evaluated by measuring absorbance at 570 nm. Results are expressed as percentage (mean ± SD from at least three experiments) compared to control.

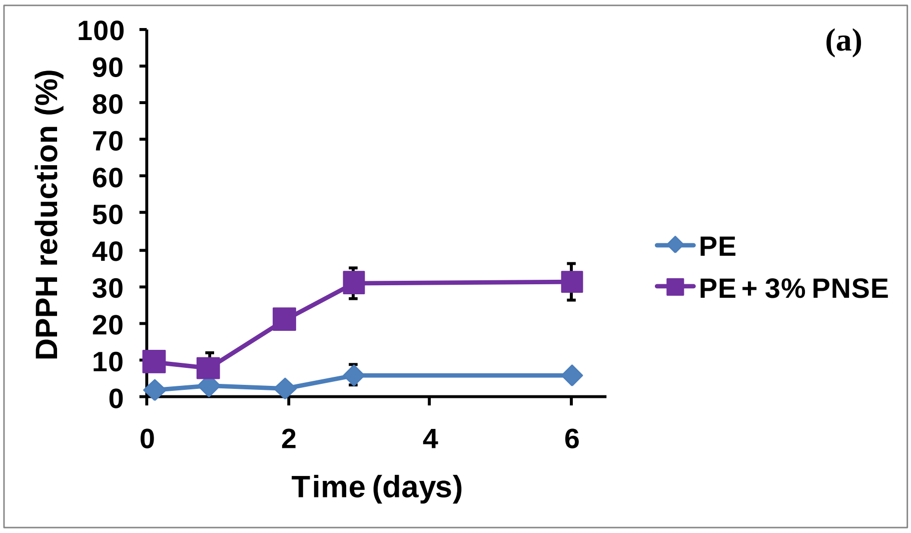

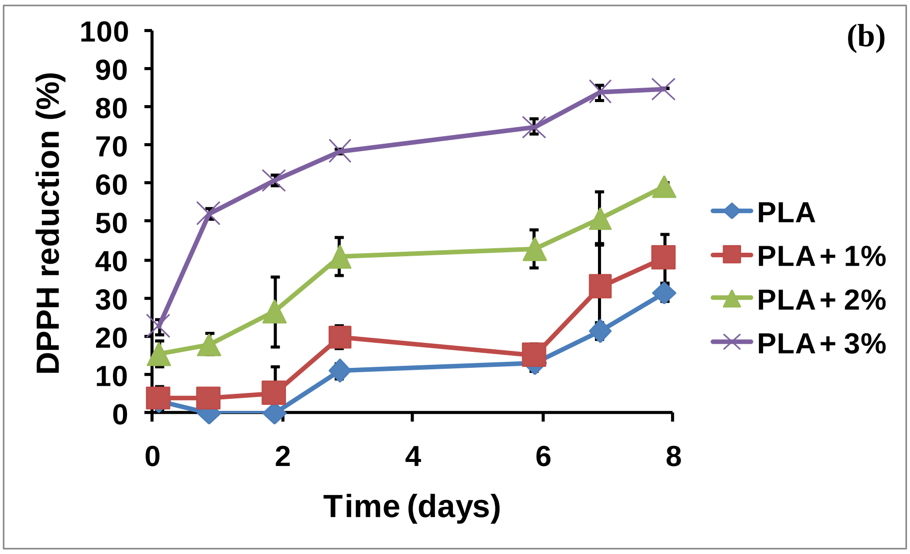

Figure S3. DPPH reducing activity of extruded (a) PE and (b) PLA films. DPPH initial concentration= 55 μM, film dose= 5 mg/mL. Reported are the mean ± SD values from three experiments.

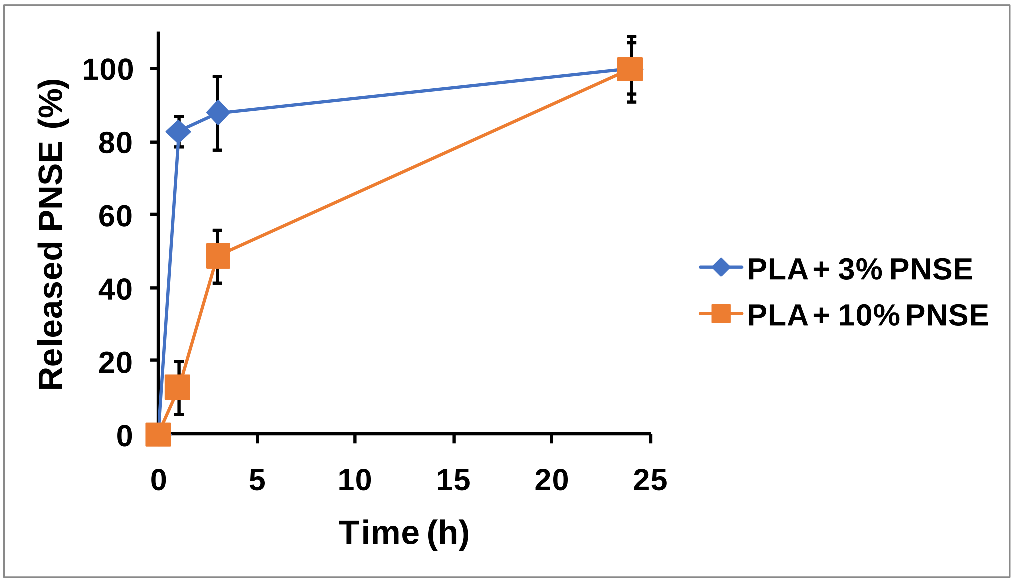

Figure S4. Release of PNSE from solvent-cast PLA films under the conditions of the DPPH assay. Reported are the mean ± SD values from three experiments.

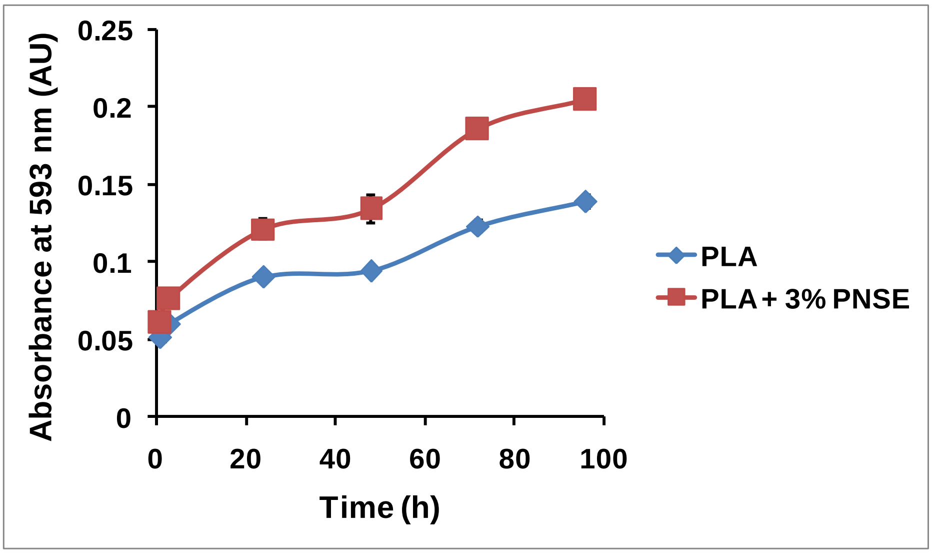

Figure S5. Fe^{3+} reducing activity of extruded PLA films. Reported are the mean ± SD values of three experiments.

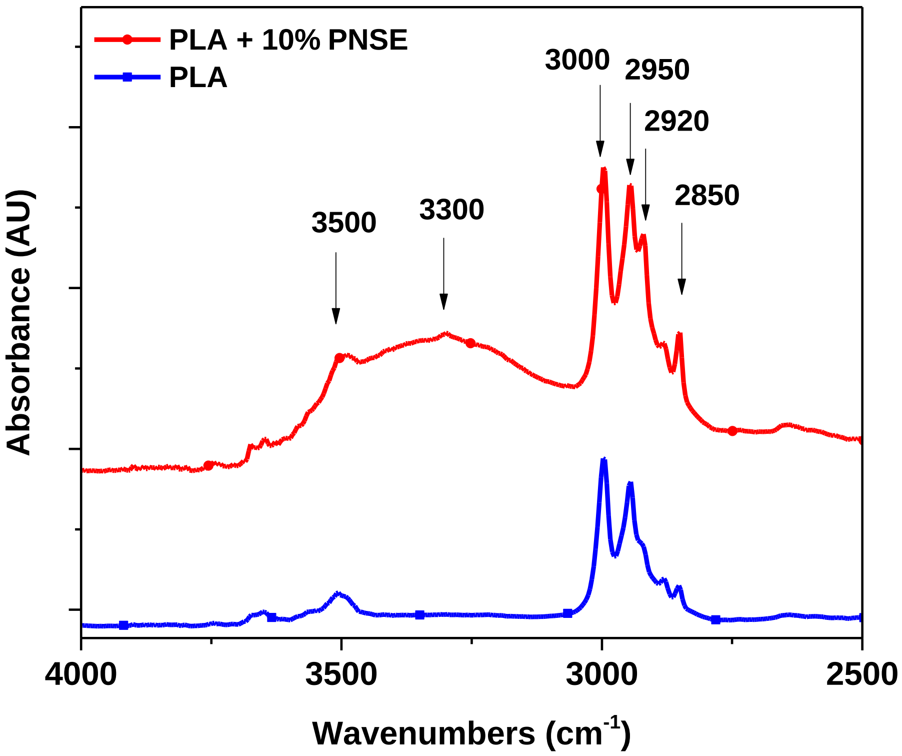

Figure S6. Close up view of the 4000-2500 cm^{-1} wavenumbers region of FTIR spectra of solvent-cast PLA and PLA + 10% PNSE films.

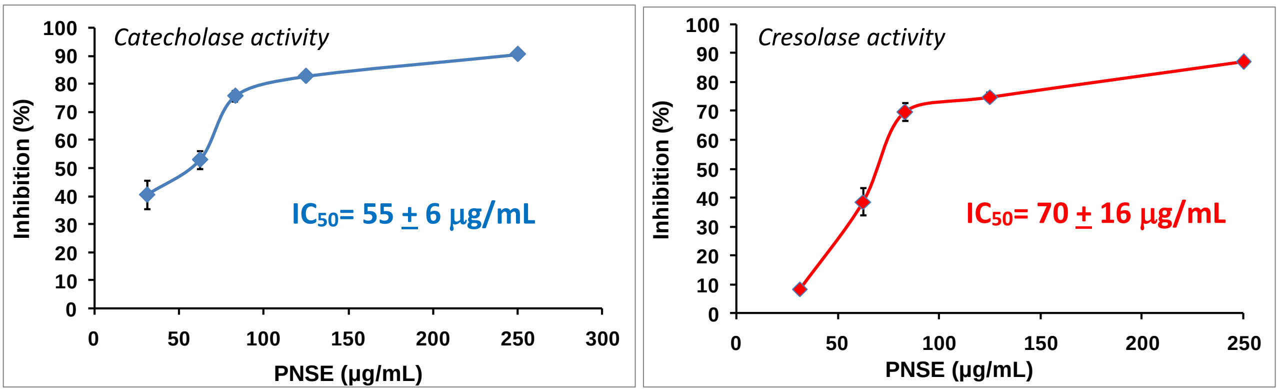

Figure S7. Percentage of inhibition of mushroom tyrosinase activity *vs* PNSE concentration using L-dopa (catecholase activity) or L-tyrosine (cresolase activity) as substrate. Reported are the mean ± SD values of at least three experiments

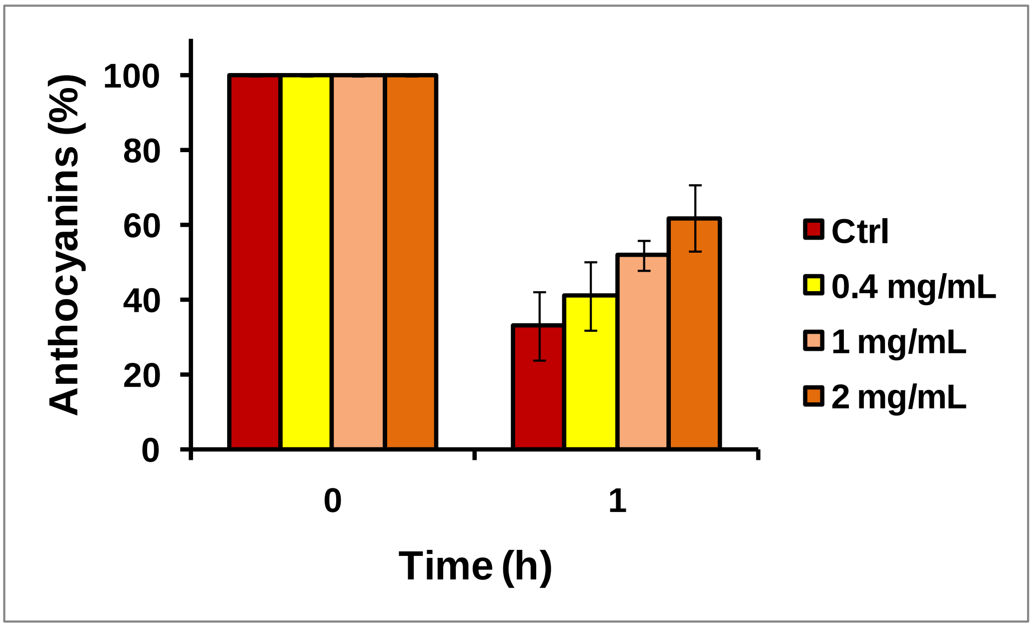

Figure S8. Effect of PNSE on anthocyanin stability at 90 °C in a bilberry juice. Reported are the mean ± SD values of three experiments.
